# Lipids and Body Mass Index in Antiretroviral-Experienced People With HIV on Doravirine-Based Treatments: A Comparison Between Dual or Triple Regimen Versus Bictegravir-Based Triple Regimen

**DOI:** 10.1155/arat/2040298

**Published:** 2025-09-25

**Authors:** Paolo Maggi, Addolorata Masiello, Barbara Menzaghi, Eleonora Sarchi, Rita Bellagamba, Giovanni Francesco Pellicanò, Filippo Lagi, Antonio Cascio, Stefania Piconi, Lucia Taramasso, Nicola Squillace, Benedetto Maurizio Celesia, Emanuele Pontali, Maria Aurora Carleo, Elena Salomoni, Sergio Ferrara, Giordano Madeddu, Goffredo Angioni, Stefano Rusconi, Salvatore Martini, Giuseppe Vittorio De Socio, Katia Falasca, Gabriella Chieffo, Olivia Bargiacchi, Deborah Fiordelisi, Giancarlo Orofino, Elena Delfina Ricci, Antonio Di Biagio, Paolo Bonfanti, CISAI Group

**Affiliations:** ^1^Department of Medicine and Surgery, University of Enna Kore, Enna, Italy; ^2^Infectious Diseases Unit, AORN Sant'Anna e San Sebastiano, Caserta, Italy; ^3^Unit of Infectious Diseases, ASST Della Valle Olona–Busto Arsizio, Varese, Italy; ^4^Infectious Diseases Unit, S. Antonio e Biagio e Cesare Arrigo Hospital, Alessandria, Italy; ^5^National Institute for Infectious Diseases Lazzaro Spallanzani Institute for Hospitalization and Care Scientific, Lazio, Roma, Italy; ^6^Unit of Infectious Diseases, Department of Clinical and Experimental Medicine, AOU Policlinico G. Martino, Messina, Italy; ^7^AOU Infectious and Tropical Diseases, Careggi Hospital, Florence, Italy; ^8^Unit of Infectious Diseases, Department of Health Promotion, Mother and Child Care, Internal Medicine and Medical Specialties, University of Palermo, Palermo, Italy; ^9^Unit of Infectious Diseases, A. Manzoni Hospital, Lecco, Italy; ^10^Infectious Diseases Unit, Ospedale Policlinico San Martino-IRCCS per l'Oncologia, Genoa, Italy; ^11^Infectious Disease Unit, Fondazione IRCCS San Gerardo Dei Tintori, Monza, Italy; ^12^Unit of Infectious Diseases, Garibaldi Hospital, Catania, Italy; ^13^Department of Infectious Diseases, Galliera Hospital, Genoa, Italy; ^14^Infectious Diseases and Gender Medicine Unit, Cotugno Hospital, AO Dei Colli, Naples, Italy; ^15^SOC 1 USLCENTRO Firenze, Unit of Infectious Diseases, Santa Maria Annunziata Hospital, Florence, Italy; ^16^Unit of Infectious Diseases, Department of Clinical and Experimental Medicine, University of Foggia, Foggia, Italy; ^17^Unit of Infectious Diseases, Department of Medicine, Surgery and Pharmacy, University of Sassari, Sassari, Italy; ^18^Infectious Diseases Unit, SS Trinità Hospital, Cagliari, Italy; ^19^Infectious Diseases Unit, Legnano Hospital ASST Ovest Milanese, Legnano, Italy; ^20^Infectious Disease Unit, University Hospital Luigi Vanvitelli, Naples, Italy; ^21^Unit of Infectious Diseases, Santa Maria Hospital, Perugia, Italy; ^22^Clinic of Infectious Diseases, Department of Medicine and Science of Aging, G. D'Annunzio University, Chieti-Pescara, Chieti, Italy; ^23^Unit of Infectious Diseases, Sant'Anna Hospital, ASST Lariana, Como, Italy; ^24^Unit of Infectious Diseases, Ospedale Maggiore Della Carità, Novara, Italy; ^25^Department of Biomedical Sciences and Human Oncology, Clinic of Infectious Diseases, University of Bari Aldo Moro, Bari, Italy; ^26^Division I of Infectious and Tropical Diseases, ASL Città di Torino, Torino, Italy; ^27^Fondazione A.S.I.A. Onlus, Buccinasco, Milan, Italy; ^28^Department of Health's Sciences, University of Genoa, Genoa, Italy; ^29^Infectious Disease Unit, Fondazione IRCCS San Gerardo Dei Tintori, Monza-University of Milano-Bicocca, Monza, Italy

**Keywords:** adverse events, bictegravir, body weight, doravirine, HIV, metabolic safety

## Abstract

**Objective:** To evaluate the lipid profile and body mass index (BMI) in antiretroviral-experienced people living with HIV (PLWH) starting therapy with two doravirine (DOR)-based regimens (dolutegravir (DTG)/DOR or lamivudine (3TC)/tenofovir disoproxil fumarate (TDF)/DOR).

**Methods:** Data from the Surveillance Cohort Long-Term Toxicity Antiretrovirals (SCOLTA) prospective database, including all experienced PLWH who started treatment with DTG/DOR and 3TC/TDF/DOR. To obtain a comparable sample, subjects on emtricitabine (FTC)/tenofovir alafenamide (TAF)/bictegravir (BIC) were matched 1:1 (by sex, age (±1 year), at least one between dyslipidemia and statin use) with those on 3TC/TDF/DOR.

**Results:** Among 355 PLWH on viral suppression, the median age was 53 years; men represented 74.9% of the sample. At baseline, 147 people treated with FTC/TAF/BIC had a better lipid profile and lower CD4 cell count than 147 people treated with 3TC/TDF/DOR; diabetes was less frequent in the latter group. After 6 and 12 months, the BMI did not significantly change in any of the groups. Total cholesterol (TC) level significantly declined in PLWH on 3TC/TDF/DOR but not in FTC/TAF/BIC and remained unchanged in DTG/DOR. LDL-C showed a similar trend, with the most marked decline in the 3TC/TDF/DOR group and no difference in FTC/TAF/BIC. The TC/HDL-C ratio declined significantly in 3TC/TDF/DOR and DTG/DOR but not in FTC/TAF/BIC. Over the entire observation period (median 18 months, interquartile range 10–30), 43 (12.1%) PLWH interrupted the cohort drug, mainly because of adverse events (*n* = 15), with 12 lost to follow-up and 12 simplifications.

**Conclusions:** The regimens were well tolerated in terms of lipid profile and BMI. Persons treated with 3TC/TDF/DOR triple regimen showed a better lipid profile, as expected, whereas those on DTG/DOR did not show any significant changes.

## 1. Introduction

The management of HIV infection has seen remarkable progress in recent decades, with antiretroviral therapy (ART) transforming what was once a fatal disease into a manageable chronic condition. However, as the patient survival improved, new challenges have emerged in relation to the long-term side effects of new treatment regimens. Among these, clinical and scientific interest is growing on metabolic tolerability and the impact on body mass index (BMI). In fact, HIV itself, as well as some ART drugs, has been associated with dyslipidemia, insulin resistance, and other metabolic disorders. These side effects can negatively affect cardiovascular health [1–5]. Additionally, changes in the BMI throughout ART can have remarkable clinical implications, as adverse outcomes can associate with both weight gain and weight loss [6, 7].

Several studies have reported that tenofovir disoproxil fumarate (TDF)–based regimens are associated with lower levels of total cholesterol (TC), low-density lipoprotein cholesterol (LDL-C), and sometimes triglycerides (TGL). This effect is often observed when compared to other nucleoside reverse transcriptase inhibitors (NRTIs) [8].

On the contrary, tenofovir alafenamide (TAF) has been associated with a less favorable lipid profile. Studies have consistently shown that initiating TAF-based regimens or switching from TDF to TAF leads to increases in TC, LDL-C, and TGL [9, 10]. The clinical significance of these changes in terms of actual CVD risk is still under investigation. Some studies suggest that despite the less favorable lipid profile, the overall cardiovascular risk may not be significantly different from TDF, especially when considering the benefit of improved bone and renal health [10].

Doravirine (DOR) is a newly approved antiretroviral, belonging to the class of non-nucleoside reverse transcriptase inhibitors (NNRTI). It is well tolerated and usually leads to an improved lipid profile in people living with HIV (PLWH) experienced to ART [11–16]. Both dolutegravir (DTG) and bictegravir (BIC) are integrase strand transfer inhibitors (INSTIs) with well-established efficacy and safety profiles, showing some differences in their side effects and metabolic management [17, 18].

NRTIs sparing regimens based on INSTIs are generally considered to have a good lipid profile. Regimens containing DTG or BIC are often associated with stable or only slightly elevated lipid levels and are often preferred for patients with pre-existing dyslipidemia or high cardiovascular risk [19], although a recent study suggested that INSTI use might be related to the risk of hypertension [20].

This study aims to compare the metabolic tolerability and impact on the BMI in experienced PLWH starting therapy with two DOR-based regimens: DTG/DOR or lamivudine (3TC)/TDF/DOR. Data were compared with those obtained from a third group treated with emtricitabine (FTC)/TAF/BIC. Moreover, data regarding adverse events (AEs) and treatment discontinuation of the three regimens were collected and analyzed.

## 2. Patients and Methods

We analyzed data from the Surveillance Cohort Long-Term Toxicity Antiretrovirals (SCOLTA) prospective database. The SCOLTA project is a multicenter observational study that started in 2002. It prospectively follows PLWH starting newly marketed antiretroviral drugs to identify toxicities and AEs in a real-life setting [21]. The SCOLTA project uses an online pharmacovigilance program and involves 25 Italian Infectious Disease Centers (https://www.cisai.it).

In brief, both ART naïve and ART-experienced PLWH can be included in SCOLTA, if they are > 18 years and agree on study entry. Clinical data collected include sex, age, ethnicity, weight, height, CDC stage, ART history, concomitant diseases, and chronic therapies other than ART. Laboratory data include HIV-RNA, CD4 +T-cell count, and biochemical data and are prospectively collected in anonymous form in a central database every 6 months. AEs are collected prospectively as soon as they are clinically observed.

In this article, we had two aims: evaluating the durability and the metabolic safety of DOR both in the 3-drug formulation and in the NRTI-sparing dual regimen DTG/DOR, comparing the metabolic safety in PLWH on the 3-drug DOR formulation with that of PLWH that were prescribed another 3-drug formulation, based on BIC(FTC/TAF/BIC). Since PLWH in the BIC cohort were younger and thus had less comorbidities and were less frequently prescribed a statin to obtain a comparable sample, subjects on FTC/TAF/BIC were matched by sex, age (±1 year), dyslipidemia, and/or statin use (at least one of these two criteria) with those on 3TC/TDF/DOR. We collected demographical information, risk factors for HIV infection, viroimmunological data, and cause of treatment interruption.

The first participant was enrolled in the BIC cohort in January 2019 and in the DOR cohort in February 2020. The enrollment is still ongoing.

Baseline was considered as T0, 6-month follow-up T1, and 12-month follow-up T2. We calculated the variation in TC, high-density lipoprotein cholesterol (HDL-C), LDL-C, TGL, TC/HDL-C ratio, weight, and BMI at T1 (T1-T0) and T2 (T2-T0).

Data were described using mean and standard deviation (SD) for normally distributed continuous variables, median, and interquartile range (IQR) and for not normally distributed continuous variables and frequency (%) for categorical and ordinal variables. Distribution normality was assessed using the graphical quantile–quantile method. Baseline differences between means were tested using the analysis of variance between medians employing the nonparametric Mann–Whitney test, and proportion comparisons were performed using the chi-square test.

Intragroup change from baseline was evaluated using the paired *t*-test. Changes from baseline between the cohorts were compared using a multivariable general linear model, including variables that were significantly different between groups, at baseline, and potentially confounding (variables reported in the footnotes) and expressed as mean and 95% confidence intervals (95% CI).

Frequencies of discontinuation for any reasons and AEs during the first year of treatment were compared using the Kaplan–Meier survival curves (and log-rank test). Reasons for interruptions were described.

All *p* levels were two-sided, at the significance level < 0.05. All statistical analyses were performed using SAS for Windows 9.4 (SAS Institute, Cary, NC).

The original study protocol was approved on 18 September 2002, and four amendments were approved on 13 June 2013, 20 December 2019, 12 May 2020, and 12 June 2023 by the coordinating center at Hospital “L. Sacco”—University of Milan, Milan (Italy), and thereafter by all participating centers. Written consent for study participation was obtained from all participants, and the study was conducted in accordance with the ethical standards laid down in the 1964 Declaration of Helsinki and its later amendments and by Italian national laws.

## 3. Results

Overall, in this analysis, 355 PLWH were included, 61 on DTG/DOR, 147 on 3TC/TDF/DOR, and 147 on FTC/TAF/BIC. The main baseline characteristics are reported in Table 1.

As regards those treated with DOR, people on dual regimen (DTG/DOR) were older and had higher proportions of comorbidities and polypharmacy. They were also switching from mainly dual regimens (50.8%), with a significantly higher proportion of PIs and lower proportion of TDF and TAF.

In the DTG/DOR group, mean LDL-C was significantly lower than that in the 3TC/TDF/DOR, which is also expected because of the highest proportion of people on statin treatment.

The FTC/TAF/BIC group was selected as the comparison for the 3TC/TDF/DOR one: they had lower median CD4 cell count and CD4/CD8 ratio. Despite being matched for age, sex, dyslipidemia, and statin use, the mean values of LDL-C and TC/HDL-C ratio were lower, and the mean HDL-Cwas higher in the FTC/TAF/BIC group, reflecting a better lipid profile at treatment start.

### 3.1. Metabolic Safety

**Figure 1:**
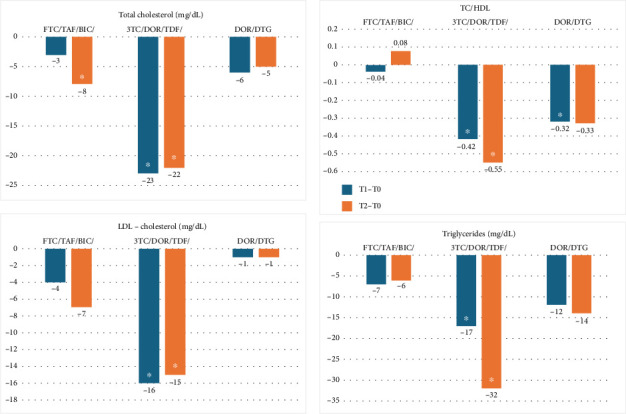
Lipid variation and weight change at T1 and T2 (^∗^change from baseline *p* < 0.05).

In Figure 1, changes from baseline are shown for TC, LDL-C, TGL, and the TC/HDL-C ratio at T1 and T2. Comparing the three regimens at the univariate analysis, we found that weight and BMI did not significantly change from baseline in any of the treatment groups, and the variation was not different among them.

In Table 2, changes from baseline are shown for weight, BMI, TC, LDL-C, HDL-C, TGL, TC/HDL-C ratio, and eGFR at T1 and T2. At the multivariate analysis, we found that weight and BMI did not significantly change from baseline in any treatment groups, and the variation was not different among them. On the contrary, TC changed significantly in PLWH on 3TC/TDF/DOR and in FTC/TAF/BIC. LDL-C showed a similar trend, with the most marked decline in the 3TC/TDF/DOR group, no significant difference in DTG/DOR, and a borderline significant decline in FTC/TAF/BIC.

The TC/HDL-C ratio significantly decreased over time in the 3TC/TDF/DOR (T1 and T2) and in the DTG/DOR (T1) groups but not in the FTC/TAF/BIC.

Repeating the multivariate analysis on the sample of PLWH not on statins (*n* = 302), we found similar results: At T1, TC declined in 3TC/TDF/DOR (−23 mg/dL, 95% CI −28 to −17) but not in DTG/DOR (−7 mg/dL, 95% CI −17 to 3) and FTC/TAF/BIC (−1 mg/dL, 95% CI −7 to 5; T2). At T2, the decline was seen in all three groups (3TC/TDF/DOR −23 mg/dL, 95% CI −30 to −16; DTG/DOR −14 mg/dL, 95% CI −27 to −2; FTC/TAF/BIC −8 mg/dL, 95% CI −17 to −2). Similar trends were also observed in LDL-C levels and TC/HDL-C ratio over time. Statins were prescribed after starting the study in two people on 3TC/TDF/DOR and one each in DTG/DOR and FTC/TAF/BIC: excluding them the findings did not change.

Finally, we examined the lipid variations in the 3TC/TDF/DOR group, comparing PLWH switching from a TDF-including regimen (*n* = 36) to those who switched from other regimens (*n* = 111). The newly introduced TDF had the expected significant effect on lipid reduction, but blood lipid parameters also improved in people who switched from other TDF-including regimen (T2: TC -12 mg/dL, 95% CI −24 to −1; LDL-C −10 mg/dL, 95% CI −20 to 0; TC/HDL-C −0.51, 95% CI −0.95 to −0.07). In the other groups, excluding PLWH switching from a TDF-including regimen (7 in FTC/TAF/BIC and 1 in DTG/DOR) did not change the mean lipid variations.

Regarding renal function, eGFR showed the expected declining trend in FTC/TAF/BIC and DTG/DOR groups, while in people on 3TC/TDF/DOR, it remained, on average, stable.

### 3.2. AEs

Overall, 15 PLWH interrupted their regimen because of AEs. The grade was specified in nine cases out of 15: eight were grade 1-2 events. In the FTC/TAF/BIC group, six AEs led to discontinuation. They were mainly central nervous system (CNS) disturbance (depression + sleep disturbance, myalgia + sleep disturbance, and agitation); excessive weight gain, itching, and renal impairment were the reasons for the other interruptions. In the 3TC/TDF/DOR group, reasons for 7 discontinuations were myalgia (2), depression (1), asthenia (1, Grade 3), nausea (1), proteinuria (1), and osteopenia diagnosis (1).

In the DTG/DOR group, two people discontinued because of Grade 1-2 events (arrhythmia, hypokalemia + confusional state).

### 3.3. Treatment Discontinuation

**Figure 2:**
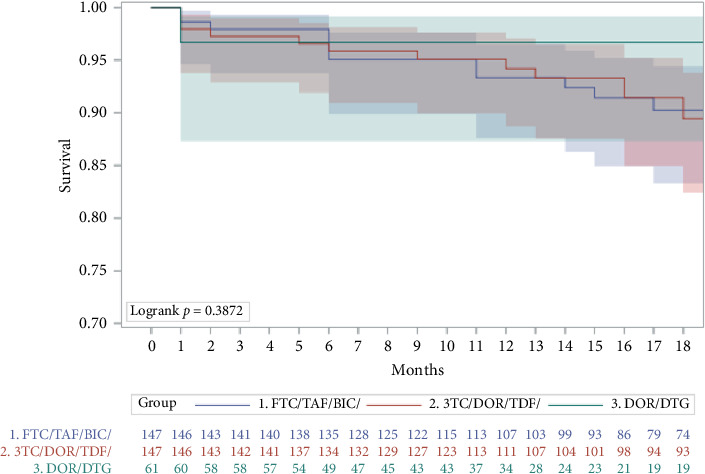
Kaplan–Meier curve for regimen discontinuation due to any reasons.

After a median observation time of 18 months (IQR 10–30), 43 PLWH discontinued their regimen. Reasons for discontinuation are reported in Table 3. The Kaplan–Meier curve (Figure 2) did not show a difference among the group in terms of discontinuation for any reasons (*p* = 0.39) or for AEs (*p* = 0.97). Truncating the observation at 1 year, the proportion of PLWH still on treatment was 93.9% in the FTC/TAF/BIC, 94.6% in the 3TC/TDF/DOR, and 96.7% in the DTG/DOR group (*p* = 0.78).

## 4. Discussion

In this study, we observed that the lipid profile improved in PLWH on DOR regimens, and that durability and viral suppression were at high levels both in the dual and triple regimens.

Patient data at baseline allow us to infer elements regarding the difference in choice of the three regimens evaluated. In fact, people on DTG/DOR dual regimen were older and had higher proportions of comorbidities and, consequently, of polypharmacy. Not unexpectedly, they were frequently treated with statins, and this could account for the significantly lower baseline LDL-C level as compared to the 3TC/TDF/DOR group. Many of them were already treated with dual regimens and shifted from a PI-based regimen, whereas few were previously treated with tenofovir DF or AF. This is probably due to the fact that this regimen represents, even if nonsingle-tablet, a valid option in older patients, with comorbidity and polypharmacy, to address problems of drug–drug interactions, drug toxicity (shifting from boosted PI regimens), and drug load (maintaining or shifting to a dual-drug regimen). This is consistent with previous observations where this regimen was preferred for PLWH at higher cardiovascular or metabolic risk, and at risk for toxicity or drug–drug interactions [22, 23].

On the other hand, the better lipid profile at FTC/TAF/BIC treatment start (lower mean value of LDL-C and higher mean HDL-C), even after matching for age, sex, and statin use, reflects the clinician's choice of a more lipid-friendly regimen (3TC/TDF/DOR) in patients with lipid abnormalities.

Regarding weight and BMI, as seen in Table 2, we did not observe significant changes from baseline in any treatment groups, and the variation was not different among them, even in the presence of a slight decrease in the 3TC/TDF/DOR group (both in T1-T0 and in T2-T0), a slight increase in the FTC/TAF/BIC group in T1-T0, followed by a slight decrease in T2-T0, and a slight increase in the DTG/DOR group both in T1-T0 and in T2-T0, worth to be evaluated in a longer follow-up. The multivariate analysis confirmed that weight and BMI did not significantly differ from 0 in each of the three groups.

Regarding lipid profile, at univariate analysis, we observed in the 3TC/TDF/DOR group a significant decline in TC, LDL-C, TC/HDL-C ratio, and TGL; in the DTG/DOR group, TC, TC/HDL-C ratio, and TGL declined significantly but not LDL-C; in the FTC/TAF/BIC TC/HDL-C ratio, LDL-C and TGL did not show significant variations, whereas TC declined at T2.

The multivariate analysis confirmed the univariate findings, although statistical significance was sometimes lost in the DTG/DOR regimen, likely because of the small size of this group.

Unexpectedly, eGFR declined in the FTC/TAF/BIC and DTG/DOR groups, while in 3TC/TDF/DOR, it remained stable. This is apparently in contrast with the well-known toxic effect of TDF on renal function [24]. However, unlike those in FTC/TAF/BIC (8%) and DTG/DOR (21%), almost 50% of the patients in 3TC/TDF/DOR came from NNRTI-based regimens, mostly consisting of rilpivirine, a modest inhibitor of proximal tubular creatinine secretion. In our opinion, this may account for a moderate and early nonprogressive creatinine-estimated eGFR reduction observed in patients previously treated with rilpivirine. Moreover, DTG is an inhibitor of proximal tubular creatinine excretion, and this could explain the greatest decline of eGFR in the DTG/DOR group. On the other hand, DOR and BIC have a neutral effect on creatinine-estimated eGFR. Further evaluations are however warranted to better clarify these findings [25].

Regarding AEs and treatment discontinuation, in our experience, the three regimens were well tolerated, with few discontinuations and no significant differences among them.

Also, these findings are consistent with previous research. In fact, in a prospective observational study, DOR plus DTG proved to be a durable treatment option even in extensively pretreated individuals [26]. In a recent retrospective, monocentric analysis, including all subjects who started a DTG-based two-drug regimen from 2018 to 2022 as a simplification, DTG plus DOR durability resulted high over a long follow-up, albeit lower than that in the other two-drug regimens. The authors suggested that this combination might be an effective option in people with HIV that has been proven difficult to treat [27]. DTG with DOR was also well tolerated in highly treatment-experienced PLWH, achieving virologic suppression in most people [28]. In the abovementioned study [22], a small cohort of elderly patients with multimorbidity and on polypharmacy switched to DTG/DOR dual regimen (mostly from boosted protease inhibitors), a viral suppression among all subjects was observed together with a statistically significant reduction in BMI, body weight, waist circumference, and serum creatinine levels. Lipid and fasting glucose values did not change significantly. In an observational, retrospective study, simplification to a single-tablet regimen of 3TC/TDF/DOR in virologically suppressed PLWH was effective and showed a good tolerability profile, in association with a significant improvement in serum lipid levels [29]. In a study on DOR efficacy and safety in adult naive or treated PLWH patients starting a DOR-based regimen mainly for toxicity and, especially, weight gain, virological suppression was maintained in 83% patients with virological control and noticed in 82% of the nonvirological control group. Moreover, renal safety and metabolic tolerance were good [23]. Even an intermittent 3TC/TDF/DOR regimen has been suggested to maintain a high level of viral suppression [30]. Recently, a study on PLWH without previous virological failure and well suppressed, who switched to DOR (mainly 3TC/TDF/DOR), compared with people who continued non-DOR-containing regimens (mainly INSTI), found similar effectiveness and safety [31].

This study has some potential limitations. First, the Infectious Diseases Clinics involved in the SCOLTA study do not formally represent the Italian Clinics at the national level because they participated in this study on a volunteer basis. Second, the study participants were representative of PLWH in need of initiating a new ART drug in the considered periods, instead of all PLWH followed in the participating Infectious Diseases Clinics. Lastly, PLWH treated with the two STR regimens still had different baseline characteristics, even though we matched them for the main factors potentially affecting their metabolic safety. Despite these limits, our study has the strength to describe a fairly large, real-life cohort of PLWH on DOR-based regimens, followed up prospectively in multiple centers across Italy, in a research network specifically designed to improve postmarketing surveillance of adverse reactions to antiretrovirals.

In conclusions, metabolic tolerability and the impact of ART on the BMI are nowadays areas of growing clinical and scientific interest. We believed we would help fill this knowledge gap evaluating the effect on these parameters in antiretroviral-experienced PLWH starting therapy with DOR-based regimens (associated with DTG or 3TC/TDF), or with FTC/TAF/BIC. In our study, the three therapeutic regimens were well tolerated in terms of lipid profile and body weight. Although the 3TC/TDF/DOR triple regimen, likely due to the statin-like effect of TDF, showed a better lipid profile; people on DTG/DOR also experienced a slight improvement. No differences in AEs and treatment discontinuation were noticed. These observations contribute to provide information to optimize the management of ART in PLWH, especially those with dyslipidemia and high BMI.

## Figures and Tables

**Table 1:** Baseline characteristics of 355 PLWH enrolled in the SCOLTA cohorts, according to the treatment group.

	FTC/TAF/BIC *N* = 147		3TC/TDF/DOR *N* = 147			DTG/DOR *N* = 61		
	*N* or mean or median	% or ±SD or IQR	*N* or mean or median	% or ±SD or IQR	*p* ^∗^	*N* or mean or median	% or ±SD or IQR	*p* ^∗∗^
Age, years, median (IQR)	52	44–58	52	43–57	0.92	58	54–62	< 0.0001
Male sex, *n* (%)	111	75.5%	111	75.5%	1.00	44	72.1%	0.75
Caucasian, *n* (%)	131	89.1%	130	88.4%	0.85	56	91.8%	0.40
BMI (kg/m^2^), mean ± SD	25.6	±4.1	26.1	±5.1	0.46	25.5	±4.4	0.46
Risk factors for HIV acquisition, *n* (%)					0.0002			0.93
Sexual	72	49.0%	107	72.8%		43	70.5%	
IVDU	32	21.8%	18	12.2%		8	13.1%	
Others or unknown	43	29.2%	22	15.0%		10	16.4%	
HBsAg positive, *n* (%) missing	12 30	8.2% 18.6%	13 16	8.8% 9.9%	0.93	1 6	1.6% 9.0%	0.044
HCV-Ab positive^§^, *n* (%) missing	38 27	25.8% 19.7%	21 14	14.3% 10.2%	0.003	13 4	21.3% 8.2%	0.087
CD4 (cells/mm^3^), median (IQR)	602	457–890	758	527–941	0.004	656	514–871	0.12
CD4/CD8 ratio, median (IQR)	0.83	0.52–1.24	0.96	0.70–1.29	0.036	0.86	0.64–1.28	0.24
Previous treatment duration, years, median (IQR)	9.5	3.1–20.1	10.0	4.8–17.2	0.32	21.3	11.5–25.4	< 0.0001
Previous regimen included, *n* (%)								
TDF	7	4.8%	36	24.5%	< 0.0001	1	1.6%	< 0.0001
TAF	93	63.3%	63	42.9%	0.0005	18	29.5%	0.037
PI	14	9.5%	17	11.6%	0.57	33	54.1%	< 0.0001
INSTI	94	64.0%	61	41.5%	0.0001	47	77.0%	< 0.0001
NNRTI	12	8.2%	70	47.6%	< 0.0001	13	21.3%	< 0.0001
Dual regimen	6	4.1%	13	8.8%	0.10	31	50.8%	< 0.0001
Hypertension, *n* (%)	32	21.8%	33	22.4%	0.89	31	50.8%	0.0003
Diabetes, *n* (%)	14	9.5%	2	1.4%	0.002	8	13.1%	0.001
Dyslipidemia, *n* (%)	35	23.8%	35	23.8%	1.00	29	47.5%	0.001
On statin treatment, *n* (%)	18	12.2%	18	12.2%	1.00	17	27.8%	0.006
On any lipid-lowering treatments, *n* (%)	22	15.0%	24	16.3%	0.75	22	30.1%	0.026
Polypharmacy, *n* (%)					0.88			0.003
0	71	48.3%	60	40.8%		13	21.3%	
1-2	43	29.2%	58	39.5%		24	39.3%	
≥ 3	33	22.4%	29	19.7%		24	39.3%	
Total cholesterol (mg/dL), mean ± SD	192	±39	203	±43	0.016	197	±51	0.35
LDL-c (mg/dL), mean ± SD	107	±36	126	±37	< 0.0001	114	±42	0.042
HDL-c (mg/dL), mean ± SD	56	±20	50	±15	0.0007	50	±17	0.90
Triglycerides (mg/dL), median (IQR)	114	85–159	120	84–173	0.57	130	97–196	0.20
Total cholesterol/HDL-c, mean ± SD	3.73	±1.32	4.36	±1.27	< 0.0001	4.27	±1.61	0.67
eGFR (mL/min), mean ± SD	88.5	±21.8	91.8	±21.6	0.18	75.8	±28.2	< 0.0001

*Note:* HBsAG: hepatitis B surface antigen; HDL-c: high-density cholesterol; INSTI: integrase strand transfer inhibitors; LDL-c: low-density cholesterol; TAF: tenofovir alafenamide.

Abbreviations: BMI, body mass index; eGFR; estimated glomerular filtration rate; HCV, hepatitis C virus; IVDU, intravenous drug use; NNRTI, non-nucleoside reverse-transcriptase inhibitors; PI, protease inhibitors; PLWH, people living with HIV; TDF, tenofovir disoproxil fumarate.

^§^4 people with detectable HCVRNA.

^∗^Comparison between 3-drug regimens.

^∗∗^Comparison between doravirine-including regimens.

**Table 2:** Adjusted mean change from baseline and 95% confidence interval, according to the treatment group.

	*N*	FTC/TAF/BIC	3TC/TDF/DOR	*p* ^∗^	DTG/DOR	*p* ^∗∗^
BMI, T1-T0	246	0.10 (−0.39–0.59)	−0.07 (−0.45 to 0.31)	0.47	0.02 (−0.45–0.48)	0.78
BMI, T2-T0	193	0.03 (−0.67–0.73)	0.04 (−0.48–0.57)	0.96	0.02 (−0.65–0.70)	0.96
Weight (kg), T1-T0	300	0.4 (−1.1–1.9)	0.2 (−1.0–1.3)	0.75	0.1 (−1.4–1.5)	0.90
Weight (kg), T2-T0	227	0.4 (−1.5–2.4)	0.2 (−1.2–1.7)	0.79	−0.1 (−2.1 to 1.8)	0.79
Total cholesterol, T1-T0	337	−3 (−9 to 4)	**−23 (−29 to −17)**	< 0.0001	−6 (−16 to 4)	0.002
Total cholesterol, T2-T0	258	**−8 (−17 to -0)**	**−22 (−30 to -15)**	0.004	−5 (−18 to 7)	0.014
LDL-cholesterol, T1-T0	311	−4 (−10 to 1)	**−16 (−22 to -11)**	0.001	−1 (−10 to 8)	0.002
LDL-cholesterol, T2-T0	242	−7 (−13 to 0)	**−15 (−21 to -8)**	0.055	−1 (−11 to 10)	0.016
HDL-cholesterol, T1-T0	318	−0 (−2 to 2)	−2 (−4 to 0)	0.15	1 (−3–4)	0.12
HDL-cholesterol, T2-T0	252	**−3 (−7 to −0)**	−2 (−5 to 1)	0.44	0 (−5–6)	0.47
Triglycerides, T1-T0	322	−7 (−20 to 6)	**−17 (−28 to -6)**	0.20	−12 (−31 to 8)	0.62
Triglycerides, T2-T0	257	−6 (−21 to 9)	**−32 (−44 to -19)**	0.009	−14 (−39 to 10)	0.23
TC/HDL-c, T1-T0	317	−0.04 (−0.12–0.20)	**−0.42 (−0.63 to −0.21)**	0.002	**−0.32 (−0.65 to −0.01)**	0.59
TC/HDL-c, T2-T0	252	0.08 (−0.20–0.36)	**−0.55 (−0.81 to −0.29)**	0.002	−0.33 (−0.76 to 0.11)	0.35
eGFR, T1-T0	343	**−3.5 (−6.3 to −0.6)**	0.6 (−2.2–3.4)	0.078	**−7.8 (−12.2 to -3.5)**	0.0005
eGFR, T2-T0	271	−3.4 (−7.3 to 0.4)	1.1 (−2.7–5.0)	0.05	−6.2 (−12.5 to 0.1)	0.03

*Note:* The multivariate models included baseline value of the variable, age, and previous regimen (TAF, TDF, or none; PI Y/N; NNRTI Y/N; INSTI Y/N). Regarding blood lipids, HCV coinfection, and statin use at baseline were also included. Age was excluded from the model for eGFR analysis because it was incorporated in the eGFR equation. HDL-c, high-density cholesterol; LDL-c, low-density cholesterol. Bold values represent *p* < 0.05 for change from baseline.

Abbreviations: BMI, body mass index; eGFR, estimated glomerular filtration rate; TC, total cholesterol.

^∗^Comparison between 3-drug regimens.

^∗∗^Comparison between doravirine-including regimens.

**Table 3:** Reason for regimen discontinuation, according to the treatment group.

	FTC/TAF/BIC *N* = 147	3TC/TDF/DOR *N* = 147	DTG/DOR *N* = 61
Observation time (months), median (IQR)	18 (11–33)	21 (12–33)	12 (7–18)
Any	23 (15.6%)	17 (11.6%)	3 (4.9%)
Adverse event	6	7	2
Simplification or switch to long-acting regimen	9	3	0
Death	1	1	0
Other	2	0	0
Lost to follow-up	5	6	1
At 1 year	9 (6.1%)	8 (5.4%)	2 (3.3%)
Adverse event	3	5	2
Simplification or switch to long-acting regimen	2	1	0
Death	0	1	0
Others	1	0	0
Lost to follow-up	3	1	0

*Note:* Adverse events: FTC/TAF/BIC (3 central nervous system, 1 weight gain, 1 itching, and 1 renal impairment); 3TC/TDF/DOR (2 myalgia, 2 renal, 1 central nervous system, 1 asthenia, and 1 gastrointestinal); DTG/DOR (1 hypokalemia and 1 tachycardia).

## Data Availability

The data presented in this study are available on reasonable request from the corresponding author due to restrictions.
